# Evaluating the level of knowledge of HIV prevention methods and associated socio-demographic factors among adolescents before and after participating in health education in Nimule town of South Sudan

**DOI:** 10.1186/s12889-025-22025-7

**Published:** 2025-02-24

**Authors:** Samuel Bojo, Gilbert Kokwaro, Ambrose Agweyu

**Affiliations:** 1 Institute of Healthcare Management, Strathmore University Business School, Strathmore University, Nairobi, Kenya; 2 Strathmore University, Nairobi, Kenya; 3 KEMRI-Wellcome Trust Research Programme, Nairobi, Kenya; 4 London School of Hygiene and Tropical Medicine, London, UK, London, England, UK

**Keywords:** Knowledge, HIV prevention, Adolescents, South Sudan

## Abstract

**Introduction:**

Adolescents in conflict-affected settings often face limited access to health information and other prevention resources, making them more vulnerable to HIV and other sexually transmitted infections. This study assessed adolescents’ level of knowledge of at least three HIV prevention methods and sociodemographic characteristics among adolescents and associated with this knwoledge before and after participating in health education in the town of Nimule, South Sudan.

**Materials and methods:**

We collected and analysed baseline and endline data from 557 adolescents aged 10–17 aged recruited from HIV-affected households in Nimule town. The surveys were conducted between December 2020 and December 2022. Assent to participate for all participants was obtained from their caregivers while additional informed consent was obtained from adolescents aged 15–17 who were considered empowered minors by the South Sudan Ministry of Health. Adolescents were then recruited into 40 peer-led health clubs and completed a three-month comprehensive sexuality education curriculum developed by the South Sudan Ministry of Health. These participants were then followed up for 24 months, and an endline survey was conducted to collect comparabel data. Binay logistic regression analysis was used to assess the level of knowledge of at least three mIV prevention methods in line with UNAIDS conceptualisation of knowledge and associated sociodemographic factors. Associations were reported using adjusted odds ratios.

**Results:**

Of the 768 adolescents enrolled, 557 were surveyed at baseline and endline with 301 (54.0%) being females and 276 (46.0%) males. The median age was 14 years (IQR: 11–16) at baseline and 15 years (IQR: 12–17) at the endline survey. The proportion of adolescents who knew at least three methods of HIV prevention increased from 465 (83.5%) at baseline to 556 (99.8%) at the endline survey. Unemployed adolescents had a 99% reduced chance of knowing at least three HIV prevention methods (aOR 0.01, 95% CI: 0.002–0.025, *p* < 0.001), whereas adolescents who self-rated their health as excellent had a 46% lower chance (aOR 0.56, 95% CI: 0.27–1.14, *p* < 0.025).

**Conclusions:**

The increase in knowledge of at least three methods of HIV prevention at the endline survey highlights the important role peer-led health education programs plays in closing gaps in HIV prevention among adolescents in conflict-affected settings like South Sudan.

**Supplementary Information:**

The online version contains supplementary material available at 10.1186/s12889-025-22025-7.

## Introduction

Adolescents in conflict-affected settings often confrint many hurdles to getting information on HIV, including other sexual and reproductive health rights, so increasing their vulnerability to HIV [1, 2]. Studies undertaken in thirty low and middle-income countries in sub-Saharan Africa revealed poor sexual and reproductive health including HIV prevention outcomes among adolescents [3]. In South Sudan where armed conflict has raged for decades creating one of the world’s worst humanitarian crisis, high levels of poverty, illiteracy and poor health systems have hindered access to HIV information, testing and treatment services [5]. In comparison to Ugandan which has increased access to HIV services a review of South Sudan’s national HIV program performance in 2021 revealed concernig level of awareness on HIV with only 35% knwoing thier HIV status and less than 50% of people living with HIV accessing lifesaving antiretroviral therapy [5, 6]. Furthermore, a bio-behavioural surveys conducted in Juba and Nimule equally provided critical awareness gaps and higher HIV prevalence of 39% and 24.0% among young sexually exploited female sex workers respectively [7]. The intersection between armed conflict, poverty and high levels of illiteracy continue to present for midable barriers to accessing HIV health information and access to other prevention services for young adolescents. However, promising results have shown that the delivery of health education and addressing behavioural, biomedical, and structural factors are effective in addressing HIV prevention gasp among adolescents [8, 9]. Against this background, the need to address the knowledge gaps becomes a priority intervention, especially in the context of post-conflict settings like South Sudan [10, 11]. The objective of this study was therefore to assess the level of knowledge of at least three methods of HIV prevention methods and their associated socio-demographic factors among adolescents in Nimule town of South Sudan.

## Materials and methods

### Study design and participation

We conducted two cross-sectional surveys at baseline and endline for a population of adolescents aged 10–17 years before and after participating in a peer-led health education delivered in health clubs. We purposively recruited adolescents aged 10–17 years from HIV-affected households to prevent transmission of HIV from those already living with the disease as a result of vertical transmission while at the same time preventing those already exposed but didn’t get HIV from acquiring it. Working with the Antiretroviral treatment (ART) clinic in Nimule Hospital, weidenitifed eligible households from treatment registers and collected them to trained research assistants who visited and enrolled them after informed consent. This procedure was repeeated until 768 adolescents from 400 households were recruited. The study population was recruited from 17 residential areas within Nimule town: Motoyo East, Motoyo West, Kololo East, Kololo West, Rock City, Melijo, Hai Kanisa, Matara, Abila, Malakia West, Malakia East, Longia, Bio II, Jeli, Nimule Central, Olikwi and Rei. Nimule town located in at the border with Uganda was suitably selected due to its high population density comprising mainly internally displaced persons (IDPs) with frequent cross-border movement. This setting presents a typical humanitarian crisis with high population movement, increased demand for health services [12]. In addition, Nimule is home to female sex workers where high HIV and Syphilis prevalence has been reported thereby posing a greater risk of infections among adolescents [7]. Adolescents and their caregivers together received a comprehensive package of case management services including but not limited to health education, screening and referral for HIV testing, household economic strengthening and parenting support. Ethical approval was granted by the Strathmore University Ethics Review Committee (SU-IERC1278/22) and the South Sudan Ministry of Health Research Ethics Review Board (MOH/RERB/24/2020).

### Sample size estimation

We used the WHO (1991) cluster sampling strategy for estimating a population proportion with specified relative precision to estimate our sample size [13]. Assuming 50% of adolescents correctly knew at least three methods of HIV prevention at baseline, a confidence level to 95% and a design effect of 2; the estimated sample size was 768 adolescents with a 5% significance level.

**Operationalization of variables**: The outcome variable “level of knowledge of at least three methods of HIV prevention” was measured using four composite indicators adapted from the UNAIDS conceptualization of knowledge [14]. These indicators included: (1) being faithful to one sexual partner (Does having only one faithful sexual partner reduce the risk of acquiring HIV?), (2) consistent and correct use of condom during sex (Does consistency and correct use of a condom during sex reduces the risk of acquiring HIV?), (3) transmission of HIV from mother to child (can an HIV-positive pregnant mother transmit HIV to her child during (a) pregnancy, (b) at birth, and (c) during breastfeeding?, (4) Dispelling myths by answering “Yes or no” to the questions (a) Can a healthy-looking person have HIV?, (b) can a person get HIV by sharing food with a person living with HIV?). Respondents’ knowledge was categorized as “No knowledge” if the adolescent didn’t identify any of the methods measured, “low-level knowledge” if the adolescent only identified 2 methods, and “high knowledge” if the adolescent correctly identified at least three methods. The sociodemographic variables collected included sex, education level, marital status, caregiver type, employment status, and self-rated health status.

### Study procedures

Adolescents were purposively recruited from HIV-affected households and enrolled into a two-year orphans and Vulnerable Children (OVC) program. The study team began by identifying and line-listing all HIV households from the ART clininc in Nimule Hospital. The line list was verified and validated by the facility in-charge and principal investigator. These households were serially numbered and a list was given to trained research assistants who booked appointments and visited these households. Using a script, caregivers were explained the purpose of the study, and inclusion and exclusion criteria and were asked to provide assent for inclusion of their adolescents aged 10–14 years. For those aged 15–17 years (emancipated minors), consent was obtained from them instead of their caregivers. Both emancipated and unemancipated minors who consented were then recruited into health clubs and completed 31 sessions of 4 modules adapted from the South Sudan Ministry of Health Comprehensive Sexuality Education.

Before the completion of the Sexuality Education program, we conducted a baseline survey in December 2020 and collected socio-demographic and HIV risk factors to assess their level of knowledge of at least three methods of HIV prevention methods. We delivered multifaceted interventions targeting both the adolescents and their primary caregivers. This included the delivery of three-month peer-led HIV risk education sessions in adolescent health clubs, positive parenting and financial literacy training for caregivers.

This ensures caregivers of these adolescents adopt positive relationships with their adolescents provide a supportive environment to adolescents and be able to support their adolescents in matters related to HIV prevention education and sexuality. At the same time, financial literacy was meant to ensure that caregivers and out-of-school adolescents participate in income-generating activities and provide for their basic needs such as food, health, and education without financial dependence. Adolescents were administered an HIV risk screening tool (Appendix 2) and those who reported having had sex or any signs of sexually transmitted infections were referred for HIV test. Equally, we then collected similar data at an end-line survey conducted in December 2022 after 24 months of follow-up.

### Data collection

We used a structured adolescent English script survey questionnaire (Appendix 1) to collect paper-based data at both baseline (December 2020) and end-line (December 2022). The questionnaire was adapted from other related studies in a South African study to assess the knowledge, attitudes, and practices of young people toward HIV prevention [15]. The questionnaire was pre-tested and validated after minor adjustments were made before data collection. Pre-testing of the questionnaire was done using a sample of 100 adolescents who were not part of the study. During the data collection, data collectors interviewed adolescents in their health clubs after obtaining their consent. Each data collector was assigned to conduct 4–5 interviews per day with the average time of each interview being 50 min. No personal data were collected. The Monitoring and Evaluation and Research Officer conducted supportive supervision and randomly selected at least 10% of completed questionnaires from each data collector to review for consistency and completeness. Inconsistent and incomplete questionnaires were returned to the respective data collectors and corrected before entering into the Excel master survey database. The data from the pre-test were not included in the analysis as the aim was only to revise the questionnaire.

### Data analysis

The outcome variable in this study was knowledge of at least three methods of HIV prevention. Both the baseline and endline data set initially captured in the Excel database were exported into STATA version 16 for analysis. We merged the baseline with the endline dataset.

#### Test for normality

Graphical (histograms), and numerical (Shaphiro Wilk test) approaches were used to test for the normality of coninous variables. Depending ontheirdistribution, continous variables were summarised as mean and median. 

##### Univariate analysis

We conducted univariate analysis for all the sociodemographic variables and individual outcome variables to check their distribution at baseline and endline. Results were presented using tables.

##### Bivariate analysis for paired data

This was conducted between observation of the outcomes collected at baseline and those at the endline to test whether they were significantly different. McNemar’s test for paired data was used to report the proportions of observations and their respective *p*-values.

##### Bivariate analysis for non-paired data

We used the chi-square test to assess the significance of each of the participant categorical sociodemographic variables and the outcome variable. Binary logistic regression was used to estimate odd ratios and corresponding 95% confidence intervals (CIs).

##### Multivariate analysis

All variables that were associated with the outcome at *p* < 0.05 at bivariate analysis were included in multivariable level analysis. A stepwise model-building approach using a binary logistic regression analysis technique was used to arrive at the final model, the Akaike Information Criterion (AIC) was used to select the best-fit model, where the model with the lowest AIC was preferred. We reported adjusted odds ratios and corresponding *p* values and 95% confidence intervals. *P* values of < 0.05 were regarded to be statistically significant.

#### Test for multicollinearity

We conducted a multicollinearity test among all the independent variables using the correlation matrix and variance inflation factor method, and for any two variables that were collinear (*r* > 0.3 or VIF > = 10) one of such variables, especially the one with higher *p*-values at bivariate analysis was excluded from the analysis.

## Results

### Participants’ sociodemographic characteristics

Of the 768 adolescents enrolled from 400 households, 211 (27.5%) of them fled to Uganda as refugees. At both baseline and endline, only 557 were surveyed and this was considered as the sample size. More than half 301(54.0%) of the adolescents were female. The median age was 14 (IQR 11–16) at baseline with over 76.0% of them enrolled in school both at baseline and end-line survey. Equally, 462(82.9%) were cared for by their parents with none reporting being married and 430 (77.2%) reported having worked for a pay in both cash in in kind (Table 1).

**Table 1:** Respondents’ sociodemographic characteristics at baseline and end-line survey (*n* = 557)

Variable	Baseline	End line
	n (%)	n (%)
Sex		
Female	301(54.0)	301(54.0)
Male	256(46.0)	256(46.0)
Age Group		
10–14 years	328(58.9)	266(47.8)
15–17 years	229(41.1)	291(52.2)
Median age (IQR) Years	14(11–16)	15(12–17)
Education		
None	7(1.3)	7(1.3)
Primary	427(76.7)	411(73.8)
Secondary	123(22.1)	116(20.8)
Tertiary	0(0.0)	23(4.1)
Owns a phone		
Yes	121(21.7)	121(21.7)
No	436(78.3)	436(78.3)
Primary Caretaker		
Parent	462(82.9)	462(82.9)
Brother/sister	39(7.0)	39(7.0)
Uncle/Aunt	56(10.1)	56(10.1)
Marital status		
Never been married	557(100.0)	557(100.0)
Employment status		
Employed	430 (77.2)	430 (77.2)
Unemployed	127 (22.8)	127 (22.8)
Health rating		
Poor	35(43.3)	199(41.6)
Good	26(32.9)	264(55.2)
Excellent	18(22.8)	15(3.1)
Household role		
Cooking	155(28%)	155(28%)
Babysitting	44(8.0%)	44(8%)
Fetching water	261(47.0%)	260(47%)
Others	97(17%)	98(18%)
Relationship with caretakers		
Father	237(42.5%)	237(42.5%)
Mother	225(40.4%)	225(40.4%)
Brother/sister	39(7.0%)	39(7.0%)
Aunt/uncle	56(10.1%)	56(10.1%)

### Participants’ level of knowledge of at least three methods of HIV prevention

Among the eight knowledge indicators assessed, the majority 465 (83.5%) at baseline correctly identified at least three HIV preventive measures (Table 2). A larger majority (413, 74.2%) correctly knew that having one faithful sexual partner minimizes the risk of HIV transmission, and 418 (75.0%) knew that consistent and accurate use of a condom each time they had sex reduced the risk of HIV acquisition. 465 (83.5%) were aware that a healthy-looking person could nevertheless have and transmit HIV. 478 (85.8%) correctly understood that sharing meals with an HIV-infected person does not represent a risk of HIV transmission. 368 (66.1%) correctly identified HIV transmission from an HIV-infected mother to her kid at birth, whereas 354 (63.6%) were aware that nursing can transmit HIV from an HIV-infected mother to her child (Table 3). In contrast, by the end of the survey, the majority of 556 (99.8%) accurately identified at least three HIV preventive measures (Table 2). Every adolescent correctly identified that having one faithful sexual partner minimizes the risk of HIV transmission, and more than 90% accurately identified the risk of HIV transmission across all variables examined at the baseline (Table 3).

**Table 2:** Level of knowledge of HIV prevention methods (*n* = 557)

Level of knowledge on HIV prevention	Baseline	End line
Doesn’t know any method	75(13.5)	0 (0.0)
Knows 1–2 methods	17 (3.1)	1(0.2)
Knows 3 + methods	465(83.5)	556(99.8)

**Table 3:** Participants’ knowledge level on HIV prevention at baseline and end line (*n* = 557)

Variable	Baseline	End line	McNemar’s *p*-value
	Yes (%)	Yes (%),	
Knowledgeable of being faithful to one sexual partner prevent transmission of HIV	413(74.2)	557(100.0)	< 0.001
Knowledgeable of correct and consistent condom use during sex reduce the risk of HIV transmission	418(75.0)	555(99.6)	< 0.001
Knowledgeable that a healthy-looking person can have and transmit HIV	465(83.5)	553(99.2)	< 0.001
Knowledgeable that HIV cannot be transmitted by the bite of a mosquito	141(25.3)	0(0.0)	< 0.001
Knowledgeable that sharing food with a person living with HIV does not pose a risk of HIV transmission	478(85.8)	557(100.0)	< 0.001
Knowledgeable that HIV can be transmitted from a mother living with HIV to her child during pregnancy	172(30.9)	549(98.6)	< 0.001
Knowledgeable that HIV can be transmitted from a mother living with HIV to her child during birth	368(66.1)	557(100.0)	< 0.001
Knowledgeable that HIV can be transmitted from a mother living with HIV to her child during breastfeeding	354(63.6)	548(98.4)	< 0.001

### Association between level of knowledge of HIV prevention and sociodemographic characteristics

At the bivariate level of analysis, knowledge of three or more HIV preventive methods was 15% higher among male participants than female participants (COR 1.148, 95% CI: 0.71, 1.855), 9% lower in the 15-17-year age range (COR 0.91, 95% CI: 0.56,1.47), and 63% higher among adolescents with secondary education (COR1.63, 95% CI, 0.65,2.10).

Unemployment (COR 0.06, 95% CI: 0.03–0.11) and self-reported health ratings (COR 1.79, 95% CI: 1.04–3.06) were both associated with lower levels of HIV knowledge among study participants, with 79% higher odds of knowledge among participants who perceived themselves to have good health and 85% lower among those who perceived excellent health (COR 0.15, 95% CI: 0.07–0.32) compared to participants who rated their current health status as poor or fair. After adjusting for age, gender, and education in the multivariate level analysis, employment status and self-health rating were still significantly associated with knowledge of at least three methods of HIV prevention. The odds of knowing at least three methods of HIV prevention were 99% lower among unemployed adolescents (aOR 0.01, 95% CI: 0.002–0.025, *p* < 0.001) and 46% lower among adolescents with good health ratings (aOR 0.56, 95% CI: 0.27–1.14). (Table 4)

**Table 4:** Associations between knowledge and participants’ sociodemographic characteristics

Variable	No knowledge	Has Knowledge	COR (95%CI)	*P*-Val	AOR (95%CI)
	*n* (%)	*n* (%)			
Sex					
Female	45(15.0)	256(85.0)	1.00	1.00	
Male	34(13.3)	222(86.7)	1.145(0.71,1.86)	0.57	
Age Category					
10–14 Years	4513.7)	283(86.3)	1.00	1.00	
15–17 Years	34(14.9)	195(85.1)	0.91(0.56,1.47)	0.71	
Education					
Primary	63(14.6)	368(85.4)	1.00	1.00	
Secondary	16(12.8)	109(87.2)	1.63 (0.65,2.10)	0.62	
Employment status					
Employed	430 (77.2)	430 (77.2)	1.00	1.00	
Unemployed	127 (22.8)	127 (22.8)	0.06(0.03, 0.11)	< 0.001	0.01(0.002, 0.025)
Self-health rating					
Poor/Fair	35(43.3)	199(41.6)	1.0	1.0	
Good	26(32.9)	264(55.2)	1.79(1.04,3.06)	0.04	0.56(0.27,1.14)
Excellent	18(22.8)	15(3.1)	0.15(0.07,0.32)	< 0.001	0.004(0.00,0.02)

## Discussion

Bearing in mind the objective of the study to assess the level of knowledge of at least methods of HIV prevention methods, this study revealed two major findings: -

*First*, we observed a higher level of knowledge of at least three methods of HIV prevention methods across all the eight knowledge indicators measured at baseline and end-line surveys. The only two indicators with a lower level of knowledge reported were knowing that a mosquito bite cannot transmit HIV and that HIV can be transmitted from an HIV-infected mother to her unborn child during pregnancy. *Secondly*, unemployment and self-health rating were the only sociodemographic factors associated with a lower level of knowledge of at least three methods of HIV prevention methods in which the odds of knowing at least three methods of HIV prevention were 99% lower among unemployed adolescents (aOR 0.01, 95% CI: 0.002–0.025, *p* < 0.001) and 46% lower among adolescents who self-rated their health as excellent (aOR 0.56, 95% CI: 0.27–1.14, *p* < 0.001).

The higher level of knowledge of knowledge of HIV prevention methods observed at baseline could be associated with the positive impact of other existing HIV awareness programs which the adolescents could have got correct information about HIV. These among others included a peer-led comprehensive HIV prevention targeting female sex workers in Nimule which has been implemented for over 10 years and could have enhanced their knowledge of HIV prevention methods. Considering that the majority of the adolescents were enrolled in school, they could have gained knowledge through the school-based comprehensive sexuality education which has been instituted since 2017 by the South Sudan Ministry of Health and Ministry of Education with support from UNFPA. This program has a strong component of HIV prevention messaging and could have increased their knowledge. Moreover, the positive influence of parents whose children were the majority in this study, health workers, peers who had already been exposed to correct HIV information, mass awareness campaigns during World AIDS Days, and social media and radio programs could have also contributed to this higher level of knowledge before the delivery of the risk education program from this study. Nonetheless, the slightly higher level of knowledge observed at the end-line survey could be associated with the positive impact of the peer-led risk education program delivered by the health clubs that supported the study. Building on the already existing body of knowledge of HIV prevention methods, the minority of the adolescents who could not correctly identify that HIV cannot be transmitted by mosquito bite and those who disagreed that HIV can be transmitted from an HIV-infected mother to her child during pregnancy might have received correct information giving every adolescent complete information on almost all the methods of HIV prevention methods. It is also important to note that trained peers were encouraged to follow and provide consistent and correct information to adolescents with incorrect information at the baseline. This ensured that peer educators could close the knowledge gap among this segment of adolescents resulting in a higher level of knowledge. However, it is important to note that other sociodemographic factors such as age, and place of employment could have also influenced the knowledge gain observed in the end-line survey. Our findings corroborate with studies conducted in Uganda in which South Sudanese refugee adolescents [16–18] who received information on sexual risk behaviours were less likely to engage in risky sex [16].

The lower level of knowledge reported among those with excellent self-health ratings was unexpected and this could be associated with inconsistencies in self-reporting which has also been reported in other studies [17]. Studies conducted in Rwanda [18] and Ethiopia [19] Have also revealed a higher level of knowledge of HIV prevention methods among adolescents. While South Sudan is a conflict-affected setting, the similarity in the findings from this study and those from other settings demonstrate the impact of risk education on adolescents’ knowledge gain irrespective of where they are implemented.

On the contrary, a low level of comprehensive knowledge among adolescents as observed in South Africa [15] indicates persistent inconsistencies in knowledge of HIV prevention methods despite increased access to HIV information in a middle-income country like South Africa. Differences in culture gender, sexual experience, and age have also been observed to influence HIV knowledge among adolescents to a greater extent [20]. While our study focused on younger adolescents from peri-urban dwellings with the majority enrolled in school, a similar study in Ethiopia involving rural adolescents irrespective of the differences in cultural and sexual experiences also found a higher level of knowledge on HIV prevention methods [21]. On the contrary, a cross-sectional study involving urban adolescents aged 15–19 in Zimbabwe also revealed a higher knowledge level [22] among urban immigrant adolescents. This observation is suggestive of the fact that such programs when delivered with fidelity have the potential to increase the knowledge level of HIV prevention methods despite variations in context and demographic factors. It is also important to note that while these findings were similar, access to HIV information and resources remains limited in South Sudan– a country recovering from prolonged civil war. The association between unemployment and a lower level of knowledge of HIV prevention methods among adolescents suggests the generalized lower level of knowledge of HIV prevention methods. Since more than half of the adolescents in our study were in school, this finding suggests that the comprehensive sexuality education program launched by the Ministry of Health and UNFPA in 2017, was also effective in complementing HIV prevention information delivered in the peer-led health clubs by the study. Although from two distinct settings, another study among adolescents from the United States also revealed that unemployment was strongly associated with poor knowledge and engagement in HIV-risk sexual behaviours.

On the contrary, our findings also were not in line with findings from Bangladesh where age and literacy improvement were associated with increased HIV prevention methods [23]. This difference could be associated with increased access to HIV information among Bangladesh adolescents as opposed to South Sudanese adolescents. Similarly, an increased level of knowledge of prevention methods was observed among older adolescents aged 15–19 years in Zimbabwe [24] and South Sudan [25]. In contrast, a study of female sex workers in Nimule found a limited level of awareness about HIV prevention methods [26]. Similarly, a study of South Sudanese refugee adolescents in the Bidibidi refugee settlement in Uganda revealed that adolescents aged 16–18 years lacked knowledge of HIV prevention and were more likely to engage in sexual behaviour than their counterparts [16]. A study on gender differences in the level of knowledge of HIV among adolescents in low- and middle-income countries revealed that boys demonstrated higher knowledge than girls [27]. A similar study in Malawi revealed a strong association between wealth and higher education to increase comprehensive knowledge of HIV prevention [28]. On the other hand, another study revealed that exposure to media was also identified to increase the odds of higher knowledge of HIV prevention as well as acquiring HIV testing services among adolescents in Uganda [29]. Our findings were also contradicted by findings from a related study in Ghana where secondary adolescents aged 15–24 years were found to have increased misconceptions such as HIV can be transmitted by handshaking, and witchcraft, and that HIV can be cured. Finally, it is important to note that while our findings were similar to a greater extent with other related studies, it is worth noting that our study differed methodologically, notable in study settings and involvement of a combination of interventions targeting both the adolescents and their primary caregivers.

### Strengths

The internal and external validity of the study was ensured by reviewing the questionnaire against other questionnaires used in similar studies to ensure that the questions were more aligned. Piloting the questionnaire, including close supervision of data collectors, ensured that the data collected was of high quality. The choice of the before and after study design and the use of appropriate statistical approaches allowed for reliable estimation of the effect of the intervention. The use of the OVC case management approach allowed for holistic recruitment of study participants, identification of needs, delivery of targeted services, and monitoring. Moreover, the delivery of multifaceted interventions provided a wide package of services to study participants, optimized impact, and retained participants in the study.

### Limitations

Our findings should be considered in light of several limitations. First of all, this study didn’t include qualitative data, and as such we couldn’t obtain an in-depth opinion on the key findings of the study. Equally, recruitment into the study was not random as the study participants were only recruited from HIV-affected households. This could have introduced bias in comparing responses from adolescents in non-HIV-affected households. There might be some measurement bias arising from participants who were lost to follow-up, and those who declined to answer some questions. Our findings might have been influenced by social desirability bias where inaccurate responses from participants could have affected our findings. Inconsistencies in self-reported adolescent sexual behaviours have also been observed in a related study [17, 30, 31]. The broad strata within our categorical variables may have led to some residual confounding effects. The study was limited in its methodological choice of quantitative methods without triangulating with qualitative findings. Further, cross-sectional data collected at baseline and end line limited the study’s ability to make inferences on causation. Lack of resources limited the collection of data at more time points. Moreover, potential biases such as respondents, recall and social susceptibility resulting in misleading findings were also possible. Moreover, we cannot also rule out the positive influence of other HIV prevention interventions targeting female sex workers and other community-based health education programs, which could have influenced the results of this study.

## Conclusions

The increased level of knowledge of at least three methods of HIV prevention methods among adolescents in conflict-affected settings like South Sudan, highlights the important role peer-led health education programs play in closing gaps in HIV prevention; hence the need for scalability.

## Electronic supplementary material

Below is the link to the electronic supplementary material.

Supplementary Material 1

Supplementary Material 2

Supplementary Material 3

## Data Availability

[The dataset named “Appendix 3- DATA_SSD_2022” supporting this study is available on the ICPSR at Workspace (openicpsr.org), reference number (openicpsr-19438)]. All supporting materials including consent forms, ethical approvals, data collection tool, and HIV risk screening tool have been attached.
