# Supplementary material for: Evaluating the level of knowledge of HIV prevention methods and associated socio-demographic factors among adolescents before and after participating in health education in Nimule town of South Sudan
Source: BMC Public Health. 2025 Feb 24;25:746. doi: 10.1186/s12889-025-22025-7 (PMC11849267; doi:10.1186/s12889-025-22025-7)
Supplement: Supplementary file 1 — Supplementary Material 1 [file 12889_2025_22025_MOESM1_ESM.pdf]

APPENDIX 3: ADOLESCENT SURVEY QUESTIONNAIRE

Introduction: Hello, my name is…………………. . I work as a research assistant with for Agency for Research and Development Initiative South Sudan. Thank you for being part of our study. Today, I'm visiting your household to ask you a few questions about how things are doing in your home today. I will ask you questions related to the health of your family members, income and education. As always, we will maintain the privacy and confidentiality of the information you and your children (10-17) will provide. Please note that children aged 10-17 will answer a separate questionnaire. I will start with you as head of the household and interview all your children aged 10-17 years. If you agree, I will then proceed to the question. Do you agree? Yes ( ) No ( )

IDENTIFICATION DATA

CHECKED BY SUPERVISOR: Signature ______________________ Date _______________ (dd/mm/yy)

SECTION 1: SOCIODEMOGRAPHIC INFORMATION

I'm going to ask some questions about yourself.

No.	Questions	Coding Categories	SKIP
**Q001**	**Record the sex of the adolescent**	Female 1<br>Male 2	
**Q002**	In what month and year were you born?	Month [__|__]<br>Year [__|__|__|__]	
**Q003**	How old were you at your last birthday?	[__|__]	
**Q004**	Have you ever attended school?	Yes 1<br>No 2	**If No: 206**
**Q005**	a) What level of school have you attended: primary, secondary, or higher?	Primary 1<br>Secondary 2<br>Higher 3<br>Don't know 88	
	b) What is the highest grade/form/year that you have completed at that level?	Less than one year completed 00<br>Grade/form/year: __ __	
**Q006**	Now, I would like you to read this sentence to me.<br>**Show the card to the respondent.**	Cannot read at all 1<br>Able to read only parts of a 2<br>sentence 3<br>Able to read the whole sentence 4	

No.	Questions	Coding Categories		SKIP
	**If the respondent cannot read the whole sentence, probe:** Can you read part of the sentence?	No card with required language: ____________________ (language)<br>Blind/visually impaired	5	
Q007	What is your current marital status?	Married<br>Cohabiting (but not married)<br>Never been married<br>Divorced or separated<br>Widowed<br>Other: ________________________	1<br>2<br>3<br>4<br>5<br>66	
Q008	As you are aware, some people accept employment where they are compensated in cash or in-kind. Others labour at the family farm or in the family company, sell goods, or run a small business. Have you done any of this in the last three months?	Yes<br>No	1<br>2	
Q009	Do you have any physical disability?<br>Observe if the adolescent has any impairment or physical disability and record	Yes<br>No	1<br>2	

No.	Questions	Coding Categories		SKIP
Q010	Are you paid in cash or kind for this work or are you not paid at all?	Cash only<br>Cash and kind<br>In-kind only<br>Not paid	1<br>2<br>3<br>4	
Q011	Do you own a telephone	Yes<br>No	1<br>2	
Q012	What role do you play in your family?	Cooking<br>Babysitting<br>Fetching water<br>Other	1<br>2<br>3<br>4	

No.	Questions	Coding Categories		SKIP
Q013	Who is caring for you	Father	1	
		Mother	2	
		Sister	3	
		Brother	4	
		Uncle/Aunt	5	
		Other: ______________	66	

--END OF SECTION—

SECTION 2: QUESTIONS ON ACCESS TO FOOD IN THE HOUSEHOLD

No.	Question	Coding Category		SKIP
Q014	Did you or any member of your household go an entire day or night without eating anything in the previous four weeks due to a lack of food?	Yes	1	**If No: 401**
		No	2	
Q015	How frequently did this occur? **Read out responses**	Rarely (1-2 times in past 4 weeks)	1	
		Sometimes (3-10 times in the past 4 weeks)	2	
		Often (more than 10 times in the past 4 weeks)	3	

--- END OF SECTION ---

SECTION 3: ADOLESCENT HEALTH AND WELLBEING

Next, I have some questions on your health and well-being.

No.	Question	Coding Category		SKIP
Q016	In general, over the past month would you say your health has been **Read out responses.**	Excellent	4	
		Good	3	
		Fair	2	
		Poor	1	

**Q017**	How frequently do you feel too ill or worn out to engage in routine activities? Do you say...?<br>**Read out responses.**	Once in a while 1<br>At least once a week 2	
**Q018**	Do you have a person in your life who you can ask for advice on how to handle a personal issue?	Yes 1<br>No 2	
**Q019**	Do you have someone in your life who can assist you with your regular responsibilities if you are ill?	Yes 1<br>No 2	
**Q020**	Do you have a partner or other close friend who loves and cares for you?	Yes 1<br>No 2	
**Q021**	Do you have a fun activity to do with someone in your life?	Yes 1<br>No 2	

--- END OF SECTION ---

SECTION 4: ADOLESCENT SEXUAL AND REPRODUCTIVE HEALTH (HIV and AIDS)

No.	Question	Coding Categories	SKIP
**Q022**	I want to talk about something else right now. Have you ever heard of the AIDS disease?	Yes 1<br>No 2	**If No: 601**
**Q023**	Is it possible to lower one's risk of contracting the AIDS virus by having a sex with an uninfected person who has no other sexual partners?	Yes 1<br>No 2<br>Don't know / Not sure 88	
**Q024**	When having sex, may people use a condom to lessen their risk of contracting the AIDS virus?	Yes 1<br>No 2<br>Don't know / Not sure 88	
**Q024**	Is it possible for someone who appears healthy to have the AIDS virus?	Yes 1<br>No 2<br>Don't know / Not sure 88	
**Q026**	Can mosquito bites transmit the AIDS virus to people?	Yes 1<br>No 2<br>Don't know / Not sure 88	

**Q027**	Is it possible to contract the AIDS virus by exchanging food with an AIDS patient?	Yes 1<br>No 2<br>Don’t know / Not sure 88	
**Q028**	Can a mother pass on the AIDS virus to her unborn child:<br>a) During pregnancy?<br>b) During childbirth?<br>c) Through breastfeeding?	Yes No DK<br>a) During pregnancy 1 2 88<br>b) During delivery 1 2 88<br>c) During breastfeeding 1 2 88	
**Q029**	Have you ever had a test to see if you are infected with the AIDS virus?	Yes 1<br>No 2	**If No: 513**
**Q030**	What was the HIV test's outcome, if you don't mind sharing?	Positive 1<br>Negative 2<br>Didn’t receive the results 3<br>Don’t feel comfortable sharing 4	**If not Positive: 513**
**Q031**	Do you use anti-retroviral or ARVs?	Yes 1<br>No 2	
**Q032**	If yes to Q3 above, how many times in the last week were you not able to take one of your ARVs pills?	Number of times ___	
**Q033**	When did the doctor last tell you about your viral load test?	Date ________<br>Don’t know___	
**Q034**	In the last month, has anyone else in your household been so sick that he/she needed to go to the hospital?	Yes 1<br>No 2<br>If yes, list names/ages ___	
**Q035**	Is the health facility in your community easy to access?	Yes 1<br>No 2	

--- END OF SECTION ---

SECTION 5: HIV RISK FACTORS AND INTENTIONS

No.	Question	Coding Categories	SKIP
**Q036 (a)**	In the past 30 days, have you experienced the following:	Genital discharge 1<br>Genital sores 2<br>Burning urination 3<br>Genital itching 4	
**Q036 (b)**	If yes to any of the above, did you seek help irrespective of the type of help?	Yes 1<br>No 2	
**Q036 (c)**	If yes to the above to above, from whom did you seek help?	Health worker 1<br>Friend 2<br>Family member 3<br>Other 4	
**Q037**	In the past 4 weeks, have you slept outside your home?	Yes 1<br>No 2	
**Q038**	In the past 4 weeks, have your parents got angry with you because you came home very late?	Yes 1<br>No 2	
**Q039**	In the past 4 weeks, have your parents got angry with you because you were with a person of your opposite sex?	Yes 1<br>No 2	
**Q040**	In the past 4 weeks, has anyone touched you on your genitals? That is your penis or vagina	Yes 1<br>No 2	
**Q041 (a)**	Have you ever had sex in your lifetime?<br>That is when a boy/man inserts the penis into your vagina	Yes 1<br>No 2	
**Q041 (b)**	If yes to the above, did your partner use a condom?	Yes 1<br>No 2	
**Q041 (c)**	Do you intend to have sex in the next 30 days?	Yes 1<br>No 2	

**Q042 (a)**	Have you ever taken any drinks that contain alcohol in your lifetime?	Yes 1<br>No 2	
**Q042 (b)**	Do you intend to drink alcohol in the next 30 days	Yes 1<br>No 2	
**Q043**	Have you ever smoked any tobacco products such as cigarettes, Shisha, Sawut, marijuana/Bangi?	Yes 1<br>No 2	
**Q044**	Are you intending to smoke any tobacco products in the next 30 days?	Yes 1<br>No 2	

SECTION 6: INTERVENTIONS RECEIVED: Now I would like to talk about services received by you or a member of your household

**No.**	**Have you or any members of your family received any of the following services in the last year?**	**Coding Categories**	**SKIP**
**Q618**	Positive parenting training and support?	Yes 1<br>No 2	
**Q619**	Cash transfer?	Yes 1<br>No 2	
**Q620**	Financial literacy and business skills training?	Yes 1<br>No 2	
**Q621**	ART treatment literacy? That session about ARVs, how they work, the benefits of correctly and consistently taking your ARVs, Viral load testing	Yes 1<br>No 2	
**Q622**	HIV risk education i.e. HIV risk identification and mitigation	Yes 1<br>No 2	
**Q623**	GBV prevention and response?	Yes 1<br>No 2	
**Q623**	Alcohol and substance use reduction?	Yes 1<br>No 2	

**Q624**	Psychosocial support counselling?	Yes 1<br>No 2	

SECTION 7: ART ENROLLMENT STATUS

No.	Question:	Coding Categories	SKIP
**Q625**	Enrolled on ART	Yes 1<br>No 2	
**Q626**	Year enrolled on ART (Type/Text):		
**Q627**	Record ART UAN		
**Q628**	In the past 90 days, have you ever missed taking your ARVs	(0= not missed, 1=Once, 2= Twice, >3 times)	

SECTION 8: COMPLETING FACILITY APPOINTMENTS

No.	Question:	Coding Categories	SKIP
**Q629**	In the past 12 months, how many total appointments were scheduled?		
**Q630**	Of the total number of appointments scheduled in the past 12 months, how many were attended?)		

SECTION 9: VIRAL LOAD/CD4 STATUS

No.	Question:	Coding Categories	SKIP
**Q631**	Did you have a viral load or CD4 count test? If no, end here	Yes 1<br>No 2	
**Q632**	If yes, record all of the dates and results of viral load or CD4 count testing starting with the oldest date. (Type)	Date:<br>Results:	

THE END OF THIS SECTION: We've come to the end of the survey. Do you have any questions to ask me? If any, please respond or refer to the health care worker. Thank you very much for your time
