# Supplementary material for: Evaluating the level of knowledge of HIV prevention methods and associated socio-demographic factors among adolescents before and after participating in health education in Nimule town of South Sudan
Source: BMC Public Health. 2025 Feb 24;25:746. doi: 10.1186/s12889-025-22025-7 (PMC11849267; doi:10.1186/s12889-025-22025-7)
Supplement: Supplementary file 2 — Supplementary Material 2 [file 12889_2025_22025_MOESM2_ESM.pdf]

HIV Risk Screening Tool

Beneficiary UIC: -……………………………………………

A) Demographics

**TOWN BLOCK:** ………………….	**RESIDENTIAL AREA**: ……………………….	**Date Risk Assessed**: DD /MM/YYYY

A) Demographics

2. Sex: Female ☐ **4. Age:**

B) Informed consent

Caregiver's Name: ______________________________

Has the Caregiver or the adolescent Accepted to be tested?: ______________________________ voluntarily give consent for my adolescent / myself to be assessed for HIV risk

Signature/Date: ______________________

Note: Parental consent is required for 15 years and below. 16+ years can consent for themselves.

C) HIV Status/Disclosure

B1. Do you know the HIV status of your adolescent?	*a) No (Proceed to Section D)*	☐
	b) Yes, the adolescent is HIV positive *(Stop Risk Assessment and mark adolescent as positive)*<br>*Is the adolescent currently on ART Yes ☐ N0 ☐ If the adolescent is not on ART refer for ART initiation*	☐
	c) Yes, the adolescent is HIV negative (Proceed to section D)	☐

D) HIV Risk assessment (section 1)

Tick where applicable;

	Yes	No
1. Did/does the adolescent have an HIV+ biological parent or sibling? If yes, refer for HIV testing and stop risk assessment. ***If no go to question 2***	☐	☐
2 Does the adolescent have ongoing risk exposure such as sexual abuse (***If yes stop risk assessment and refer for testing), If no proceed to E and F***.	☐	☐

E) HIV Risk Assessment for Adolescents (15-24years) HIV Risk assessment (section 2)

(Screen all adolescents in private setting and refer systematically for HIV testing if sexually active)

**If yes to any of the following, questions refer for HIV test**	**Yes**	**No**
1. Have you ever experienced any recurring skin problems? (Probe for HIV-related skin problems)		
2. Do you have one or both parents deceased due to AIDS-related illness?		
3. Have you been too sick to participate in daily activities in the last 3 months?		
4. Have you been diagnosed with or treated for TB?		
5. Has anyone forced you to have sex?		

**HIV Screening**	**If yes to any of the following, questions refer for HIV test**	**Yes**	**No**
1. Have you ever been paid for sex in cash or kind (goods and other favors) ?			
2. Have you had sex in your lifetime?			
3. Did you use a condom the last time you had sex?			
4. Have you or your sexual partner(s) had other sexual partners in the past 12 months?			
5. Are you currently pregnant or considering to be pregnant in the next 12 months?			
6. Do your private parts sometimes itch, burn, smell or look strange (genital discharge)? (Probing for STIs)			

F) Screening results

1. Is the adolescent at risk? Yes ☐ No ☐

If yes refer for HIV Testing. If No Mark as "test not required"

G) Referral

Has the adolescent been referred for HTS Yes ☐ No ☐

If yes, date of referral: DD/MM/YYYY Date referral completed: DD/MM/YYYY

Status disclosed? Yes ☐ No ☐ If yes HIV+ ☐ HIV- ☐
